# Knowledge, Awareness, and Prevention of Hepatitis B Virus Infection Among Korean American Parents

**DOI:** 10.1007/s10903-017-0609-1

**Published:** 2017-06-21

**Authors:** Sarah Hyun, Seulgi Lee, William R. Ventura, Joseph McMenamin

**Affiliations:** 1 Lehigh University, Bethlehem, PA USA; 2 Holy Name Medical Center, Teaneck, NJ USA; 3 St. Joseph’s Regional Medical Center, Paterson, NJ USA; 4 Virginia Commonwealth University School of Medicine, Richmond, VA USA

**Keywords:** Chronic hepatitis B, Korean Americans, Parents, Children, Health education, Health literacy

## Abstract

Hepatitis B (HB) affects 240 million people around the world, and children and young adults make up a large proportion of the infected population. Approximately 1 million people die from HB each year. Despite the seriousness of HB and its complications, many are poorly linked to clinical care. A lack of health literacy may be a critical barrier hindering access to HB care for adults as well as children in these populations. We, therefore, performed a survey to assess the level of knowledge of HB among Korean American parents. The survey was conducted on 521 Korean American adults who attended community-based HB awareness campaigns held at various locations throughout the metropolitan New York area between January 2015 and November 2016. Of these, 296 parents, who had children between ages 1 and 30, were identified. All participants were asked a series of questions regarding various aspects of HB and were evaluated on the basis of their awareness on each subject. A separate questionnaire was also employed to obtain demographic characteristics of the participants. The study revealed a significant deficit of knowledge of HB in most aspects the survey evaluated. Although the majority of the participants knew that HB is a liver disease, and many of them had been screened for HB, they had a poor understanding of vaccination, screening, their own HB status, modes of HBV transmission, and the consequences and treatment of HB. The participants also had a low level of awareness of their own children’s HB status. This study demonstrates a low level of knowledge of HB among Korean American parents electing to attend a hepatitis education program. In addition, many parents are not aware of their children’s screening and immune (or non-immune) status. The lack of health literacy may contribute to poor health access in HB care, not only in adults but also in children. This suggests an urgent need for education on HB in Korean American parents as well as in young children.

## Introduction

Hepatitis B (HB) is the most common infectious disease of liver affecting an estimated 240 million people worldwide. Approximately 1 million people die each year from HB and its complications such as liver cancer and cirrhosis [1–3]. HB is also an important disease of ethnic inequality as there is a marked disparity among various ethnicities and regions of the world in the prevalence of HB. For instance, the prevalence of HB virus (HBV) infection is highest in sub-Saharan African and East Asia, where up to 10% of the population is chronically infected. On the other hand, at 0.2%, HBV prevalence is lowest in Western Europe and North America [4, 5]. HBV prevalence varies substantially among different ethnicities within the United States, however, disproportionately affecting immigrants from HBV-endemic countries [5, 6]. Given that 40,000–45,000 people legally enter the United States every year from intermediate to highly endemic (i.e., HBV prevalence >2%) countries, HB remains a serious public health issue in this country [7]. Consequently, the US Department of Health and Human Services (HHS) introduced “Action Plan for the Prevention, Care and Treatment of Viral Hepatitis” in 2011 to provide a roadmap for better outreach, knowledge and awareness, and clinical services [8, 9].

In endemic areas, HBV transmission takes place predominantly through mother to child transmission (MTCT) at birth and through early childhood years, causing infection from early stages of life. In contrast, HBV transmission in the Western world occurs mostly during adulthood through sexual activity and intravenous drug abuse [10, 11]. The consequences of HBV infection depend on the time of infection in that 90% of perinatally infected individuals remain chronically infected, often developing chronic complications, while fewer than 5% of those infected in adulthood remain chronically infected with a lesser degree of complications [10, 11].

With the advent of the HB vaccination in the 1980s, HBV prevalence has markedly decreased [10, 13]. Prenatal screening of pregnant women also identifies chronically infected mothers and thus helps prevent potential transmission of HBV to their newborns with HB immune globulins (HBIG) and HB vaccine [12, 13]. There are still several reasons, however, why numerous children remain infected and suffer chronic complications in their latter lives. First, many children, especially among underprivileged immigrant populations in the United States and throughout the world, miss HB vaccinations. Second, because universal screening is not always accessible, many of these chronically infected children often go unnoticed to their adulthoods. Third, a large number of pregnant women are not properly screened, so their newborns often get infected through MTCT. Fourth, as HBIG is not readily available in underdeveloped countries, many of the infants born to infected mothers are at a greater risk [11, 14]. Last but not least, HBIG and HB vaccination are not 100% effective- a substantial number of children remain infected despite the vaccinations [15].

Aside from screening and vaccination as preventive measures, education is critical in ensuring that HB is not overlooked in at-risk populations [9]. The Institute of Medicine and the Centers for Disease Control and Prevention, for instance, have recognized the need to work with key stakeholders such as the government, healthcare organizations and educational institutions to develop effective education programs to increase HB awareness in at-risk populations [9]. There is still a significant lack of effort in educating the affected communities about HB, however. The absence of HB education in middle and high school health education curricula is a good example. HB education is not included in the required health education curriculum in the states of New York and New Jersey, where we find a large population of immigrants and their children from HBV-endemic countries. The schools in NY and NJ require their students to bring HB vaccination or antibody titer records, but they do not provide any education on the topics of HB prevention or management [16, 17].

Earlier studies have assessed levels of knowledge of HB in high risk populations. These investigators have demonstrated that HB knowledge in Asian Americans was low and was closely associated with demographic and acculturation factors. These and other studies have contributed to addressing health literacy and its importance in enhancing linkage to care in at-risk communities [18–20]. The current study attempts to evaluate a group of parent participants from an HBV endemic community with regard to their general knowledge of HB as well as on their level of understanding of their children’s HB status. The results suggest that general knowledge of HB in parents in an endemic population may be suboptimal and indicate a serious need for education on HB in the affected communities.

## Methods

### Study Design and Participants

This study is a community-based survey designed to assess the level of knowledge of HB in Korean American parents. The survey was taken during community HB awareness campaigns in the metropolitan New York area between January 2015 and November 2016. These campaigns, which consisted of educational seminars and screenings, were held for HBV-infected individuals, their families and other members of the community. The surveys were conducted by Youth against HB (YAH), a non-profit organization working to promote screening and access to care for children in high risk populations. The major goals of YAH are to: (A) raise awareness of HB and its complications among children throughout the world; (B) facilitate screening for chronic HB (CHB) and reduce transmission in high-risk populations by vaccination; and (C) campaign among youth populations, schools and community organizations to mobilize resources for HB care.

The survey, which was anonymous and voluntary, was conducted within the designated sites during the campaign utilizing ‘traditional paper and pencil self-administration’ methods. The survey process itself was structured to encourage participation. All participants were given education materials on HB and were advised that their participation would contribute to everyone’s healthcare in their community. Researchers handed paper questionnaires to attendees in community campaigns in person and asked them to return the completed survey to the researchers on site. The researchers helped clarify any ambiguity in the questions and motivated respondents to answer the questions.

The survey, consisting of a set of eight questions in both Korean and English, was distributed to 680 attendees, from whom 521 completed surveys were collected. The ages of the 521 ranged from 21 to 85 with a mean age of 54. More than 98% of the participants were born in Korea and preferred to communicate in Korean. Of these 521 participants, 296 people who had children aged 1–30 were identified for data analysis. All 296 parent participants were of Korean ethnic origin and had lived in the United States for a mean duration of 20.4 years.

### Data Collection and Analysis

The survey questionnaire included demographic questions and a set of eight questions specifically aimed at evaluating the respondents’ knowledge of various aspects of chronic HB, including the respondents’ awareness of their own and family members’ HB status, transmission, vaccination, and the consequences and treatment of chronic HB. Of the total 521 participants, only the data from 296 people who had children aged 1–30 were analyzed. The specific questions employed to assess participants’ knowledge are summarized in Table 1. In addition, two child-specific questions were asked: (1) Have your children been screened for hepatitis B? (Yes/No/Do not Know) and (2) Have your children been vaccinated for hepatitis B? (Yes/No/Do not Know).

**Table 1:** Questions employed to evaluate knowledge on hepatitis B

	Areas of questions	Specific questions
1	General knowledge: screening, vaccination, chronic infection	1. Are you aware of hepatitis B as a liver disease? –Yes/No 2. If I am vaccinated, I never need a screening test –True/False/Do not know 3. All the individuals infected with HBV remain infected for the rest of their lives –True/False/Do not know
2	One’s own hepatitis B status	4. Have you ever been screened for hepatitis B? –Yes/No/Do not know 5. Are you (a) immune from past infection; (b) immune from vaccination; (c) infected with HBV; or (d) not certain (do not know)?
3	Modes of transmission	6. HBV can be transmitted through the following route(s) a. Blood/sharing needles b. Sexual contact c. Birth (delivery) d. Sharing utensils
4	Consequence and treatment of infection	7. HBV can cause cancer –True/False/Do not know 8. There is a treatment for chronic hepatitis B –True/False/Do not know

Demographic questions inquired about age, gender, education, duration of residence in the United States, having primary care physicians, and source(s) relied on to obtain healthcare information.

#### Data Analysis

Descriptive statistics were used to describe the basic features of the data. Analysis was performed to compute the numbers and percentages, and means with standard deviations for certain categorical variables.

#### Ethical Approval

All procedures performed in these studies were in accordance with the ethical standards of the institutional and/or national research committee. This study was formally approved by Investigative Committee on Clinical Research (ICCR), IRB of Holy Name Medical Center, Teaneck, NJ.

## Results

### Demographics

Table 2 shows the demographics of 296 participants. There were 136 males and 160 females with a mean age of 50.1 ranging between 31 and 69 (Table 3). The mean duration of residence ± standard deviation (SD) in the United States was 20.4 ± 9.8 years. A total of 147 (49.7%) participants had primary care physicians, whom they saw on a regular basis.

**Table 2:** Demographics of all participants by gender

Gender	Total number of participants (n = 521)	Age	Number of years in the US	Chronically infected	Has PCP
Male	136	51.3	20.7 ± 9.3	20 (14.7%)	71
Female	160	49.1	19.6 ± 9.2	15 (9.4%)	76
Total	296	50.1	20.4 ± 9.8	35 (11.8%)	147 (49.7%)

**Table 3:** Demographics of the participants by age group

Age groups	Number of participants	Number of years in the US	Chronically infected	Has PCP
30–39	31	14.3 ± 6.1	6 (19.4%)	12
40–49	111	16.8 ± 7.6	15 (13.5%)	52
50–59	105	20.6 ± 9.5	10 (9.5%)	54
60–69	49	28.7 ± 11.8	4 (8.2%)	29
Total	296	20.4 ± 9.8	35 (11.8%)	147 (49.7%)

The proportion of HBV infected participants in this survey study was 11.8%, which was significantly higher than the reported prevalence for Korean Americans [21, 22]. The highest rate of HBV infection was noted in those aged between 30 and 49. Such a high prevalence rate noted in this survey may have been related to the nature of the HB awareness campaigns, which attracted a large number of individuals infected with HBV.

Low literacy has been shown to be associated with low health literacy and poor health [23, 24]. Thus, we investigated the levels of education attained by participants. We obtained data on the highest level of education from 91 participants (mean age 49.1), which revealed the following: 14 (15.4%) had high school diplomas; 56 (61.5%) had college diplomas; and 21 (23.1%) had done postgraduate work as their highest level of education (Table 4). Owing to the data’s limitations, we were not able to directly correlate educational levels with the HB knowledge of each participant.

**Table 4:** Highest level of education attained by participants

Highest level of education	Total n = 91
High school diploma	14 (15.4%)
College graduate	56 (61.5%)
Postgraduate	21 (23.1%)

### General Knowledge of Hepatitis B

In evaluating the participants’ knowledge of HB, we asked a series of eight different questions to all participants. As shown in Table 5, general awareness of HB as a liver disease was high. 276 participants (93.2%) knew of HB as a liver disease. They had a low level of understanding of screening, however, and of the lifelong outcome of HBV infection. Only 38.9% correctly understood that vaccination cannot replace screening, and that screening is always needed. The rest (61.1%) either did not comment or wrongly felt that screening was not needed once vaccination had taken place. Similarly, only 28.3% knew that HB infection does not always lead to lifelong infection.

**Table 5:** Participants’ response to eight item questionnaire

Parent participants (n = 296)	Specific questions	Aware or answering correctly
1	Are you aware of hepatitis B as a liver disease? –Yes/No/Do Not Know	276 (93.2%)
2	If you received vaccination, you do not need a screening test –Yes/No/Do Not Know	115 (38.9%)
3	All the individuals infected with HBV remain infected for the rest of their life –Yes/No/Do Not Know	84 (28.3%)
4	Have you ever been screened for hepatitis B? –Yes/No/Do not know	222 (75%)
5	Do you know if you are immune, infected with HBV, or at risk?	174 (58.8%)
6	Do you know if HBV may be transmitted through blood/needle sharing	144 (48.6%)
	Sexual contact	69 (23.3%)
	Birth; and/or	61 (20.6%)
	Sharing utensils?	85 (28.7%)
7	HBV can cause cancer –Yes/No/Do Not Know	213 (72.0%)
8	There is a treatment available for chronic hepatitis B –Yes/No/Do Not Know	159 (53.7%)

### Awareness of One’s Own Status

When they were asked about their own screening status, a majority (222 participants, 75%) were aware. On the other hand, 41.2% of the participants reported that they did not know if they were immune, infected, or at risk. Thus, despite the high screening rate, many people were unaware of the results of their screening.

### Knowledge on Transmission, Consequence, and Treatment

The participants’ knowledge about the modes of HBV transmission was low. Only 48.6, 23.3 and 20.6% of the participants, respectively, were aware that HBV could be transmitted through blood/infected needles, sexual contact, and delivery (Table 5). Further, 28.7% falsely thought that HBV could be transmitted through sharing of utensils. Participants’ knowledge of the consequences and treatment of HB was also poor. Of 296, 83 participants (28%) were unaware that HB can potentially cause cancer, and as many as 137 (46%) were unaware that antiviral treatment for HB is available.

### Parents’ Awareness of Children’s Status

In order to assess parents’ awareness of their children’s HB status (immune, at risk, or infected), we asked the parents if they knew about their children’s screening and vaccination. Only 52.7% of the parents were certain that their children had been screened for HB. The rest of the parents responded either that their children had not been screened (22.6%) or that they were not certain (24.7%). Regarding their children’s vaccination history, only 160 parents (57.1%) replied that their children received vaccination (Table 6).

**Table 6:** Parents’ awareness on children’s status

	Questions	Parents’ responses (n = 296)		
		Yes	No	Do not know
1	Have your children been screened for hepatitis B?	156 (52.7%)	67 (22.6%)	73 (24.7%)
2	Have your children been vaccinated for hepatitis B?	169 (57.1%)	30 (10.1%)	97 (32.8%)

### Sources of Health Information

Easy access to and availability of health information are critical for health literacy. In order to evaluate the participants’ health information seeking behavior, participants were asked to choose the most reliable source of information for their healthcare from the following list: Doctor, TV/Radio (in Korean or English), Newspapers/magazines (in Korean or English), Internet (in Korean or English), Family/Friends, and Community centers. 119 participants (40.2%) chose doctors as their first choice of resource. This was followed by TV and Radio (26.7%), Internet (12.8%) and Newspapers (8.4) all in Korean. Internet, TV and Newspapers in English totaled 7.4% and only a small percentage (4.4%) of respondents relied on community centers and family/friends as their first choice to obtain information on their healthcare (Table 7).

**Table 7:** Most important information source to make decisions about healthcare

Health information source	Percentage (n = 296)
Doctor	119 (40.2%)
TV, radio (in Korean)	79 (26.7%)
Internet (in Korean)	38 (12.8%)
Newspaper and magazine (in Korean)	25 (8.4%)
Internet (in English)	10 (3.4%)
Family/friends	8 (2.7%)
Newspaper/magazines (in English)	6 (2.0%)
TV (in English)	6 (2.0%)
Community center	5 (1.7%)

## Discussion

Chronic HB is an important disease of ethnic disparity in the United States affecting various minority populations disproportionately. While 2 million Americans are chronically infected with HBV, over half of these are Asian Americans and Pacific Islanders [9, 14]. As a major mode of HBV transmission in Asian populations is MTCT, a significant proportion of the infected population may be children and young adults.

There are two key issues concerning HB. First, a majority of the individuals infected with HBV are not aware of their infection, suggesting a greater need for screening in high risk populations [4, 14, 25]. Second, only a small portion of HBV infected people who require treatment are currently under care [14, 26]. The reasons for this lack of screening and linkage to care are multi-factorial and include poor health literacy and other barriers related to financial, language, and cultural issues [4, 27].

### Lack of Health Literacy on Hepatitis B

Health literacy refers to one’s ability to obtain, understand, and apply health information and services to engage in self-care and navigate the healthcare system [28]. Low health literacy serves as a potential mediator of health disparities in various racial and ethnic groups. Specifically, a number of studies have directly implicated a lack of health literacy in hindering access to HB care [18–20].

The current study demonstrates a significant lack of health literacy on HB among Korean Americans, specifically a parent population with children aged 1–30. On the other hand, education level of the 91 participants we assessed in this study showed a high average level of education, including 77 (85%) college graduates (Table 4). This may represent the average level of education in Korean immigrants as noted in a report in 2013, which showed that 52% of Korean immigrants (ages 25 and over) had a bachelor’s degree or higher [29]. Given the relatively high level of education in the participants in our study, however, these findings suggest that health literacy may not necessarily correlate with general literacy skills or with education level.

### Awareness of Children’s Status

This study is unique in that it focuses on parent participants within an ethnic community of Koreans as well as their knowledge of their children’s health status. Overall, the parents’ awareness of their own children’s HB status was poor. Only about half of the parents were certain that their children had undergone screening for HB. About a quarter (22.6%) of the participants replied that their children did not undergo screening, which would be acceptable if their children had received vaccination at birth and had mothers who were not chronically infected. Another quarter of the participants did not know whether their children had received screening. Furthermore, 33% of the participants did not know if their children had been vaccinated, and 10% denied that their children had received vaccination (Table 6). Such a meager level of awareness among parents of their children’s HB status may be reflected in poor health outcomes of their children. Indeed, previous studies have shown that low parental health literacy correlates with worse health outcomes in young children [28, 30]. Considering that over 21 million parents in the United States may have low health literacy [28], it is concerning how harmfully the parents’ low health literacy could affect health outcomes of their children.

There is very little, if any, research focusing on parents’ HB knowledge and particularly on the link between parental knowledge and children’s liver health. Previous studies evaluating the correlation between parental knowledge and children’s immunization completeness, however, have demonstrated a positive association between parental knowledge and their children’s immunization rates [31, 32]. These are also congruent with an Italian study on mothers [33], which showed that mothers’ lack of knowledge of vaccination was an important reason for failure to complete an immunization schedule.

### Sources of Health Information

People’s health literacy depends on various factors including the sources of the information they look into. The quality of the health information found can be crucial in making appropriate health decisions. Our results showed that doctors were the most reliable source of health information, and over 95% of respondents specified Korean-speaking doctors as their first choice. The second most popular source was Korean language TV and radio, followed by internet and newspaper. The respondents’ low level of knowledge on HB may then reflect a lack of emphasis on HB education through doctors and Korean media. Furthermore, limited English proficiency among the participants may also be responsible for their lack of health literacy [29]. On the other hand, it should be understood that even if the information is provided, many participants might not be able to grasp the meaning of the information correctly.

Numerous factors such as racial, cultural, language, literacy and socio-economic differences play a role in determining how one accesses health information [34]. In our study, an overwhelming majority of the participants preferred to obtain health information from sources available in the Korean language. Previous investigations have shown that Korean immigrants’ education levels were relatively high when compared with other ethnic groups, but that many of them had difficulty with English [35]. It was also reported that 53% of the Korean immigrants had limited English proficiency, compared to 50% of the total foreign born population [29]. We did not assess English proficiency in our participants, and thus we cannot say whether level of English proficiency is associated with appropriate access to health information relevant to HB care. It is well established, however, that difficulties in communication can affect health information-seeking behavior. A recent study of 1605 Latino patients with diabetes mellitus, for instance, demonstrated that those patients who lacked English proficiency may end up with poorer care [36]. Further, the lack of English proficiency may affect the respondents’ ability to oversee their children’s curricula and educational activity in schools, specifically, health education.

### HB Education in US Schools

High school health education curriculum generally includes education on sexual health, HIV/AIDS, alcohol, tobacco, and drugs [16, 17]. Education on HB is not offered even in the districts heavily populated with ethnic groups immigrating from HB endemic regions of the world. Both public and private schools (elementary, middle and high) in the states of New York and New Jersey require their students to bring vaccination or antibody titer records on HB, but their curricula do not mention the risk and basic management of HB, so they do not appear to provide education on such topics. Since Asians, Pacific Islanders (API) and African immigrants have a higher risk of having HB, for instance, schools with high populations of students from these ethnic groups should consider offering education on HB.

Lack of HB education in schools that are heavily populated with children of parents who have immigrated from HB endemic countries is an important issue of health inequality. Given the poor HB literacy among immigrant populations as seen in the current study, HB education for their children is critical. Future work may be needed on collaborating with schools, community organizations, and policy makers to develop educational programs for young children at high risk.

### Limitations of the Study

There are several important limitations to this study. First, the participants in this study may not represent the overall Korean American or other ethnic population at risk for HB in the United States. HBV prevalence among the participants in this study, for instance, was significantly higher than the HBV prevalence reported for Korean Americans in other studies [20–22]. In the current study, the participants were recruited from HB awareness forums and campaigns. As a result, these campaigns attracted many individuals chronically infected with HBV. The education and socio-economic classes may also vary among the participants, potentially affecting the outcomes of their knowledge levels on HB. Second, all the survey data were based on a self report questionnaire, potentially raising a set of issues [37]. The accuracy of the data, for instance, may rely on the participants’ honesty, memories, and their introspective ability to respond to the questions correctly. Although participants could have misunderstood the questions, this is unlikely because the questions were concrete and not abstract, and were posed in both English and Korean. In addition, there could have been response bias where participants may have felt socially pressured or obligated to answer questions, leading to untruthful answers. Third, distribution of the participants in all age groups was not even. Thus, we may not have been able to directly compare age groups in regard to their responses. Nevertheless, the current study helps demonstrate a significant lack of awareness of HB in a specific community within an endemic population, and may be applied to designing similar studies among other Asian American groups in other parts of the country, as well as in other ethnic groups.

In conclusion, our work suggests a significant lack of knowledge of HB among Korean American parents. The parents’ level of awareness of their children’ HB status was also suboptimal, potentially causing adverse health outcomes in children. Given the high rate of HBV in certain populations, more effort and resources must be devoted to educating the affected community and children on HB and its serious complications, thereby improving health disparities for racial and ethnic minorities.

#### Authors’ Contributions

SH conceived of the study, participated in the design of the study, and drafted the manuscript. SL participated in the design of the study and performed statistical analysis. JM and WRV participated in the study coordination and helped to draft the manuscript. All authors read and approved the manuscript.

#### Funding

This study was funded by a grant from Gilead Foundation.
